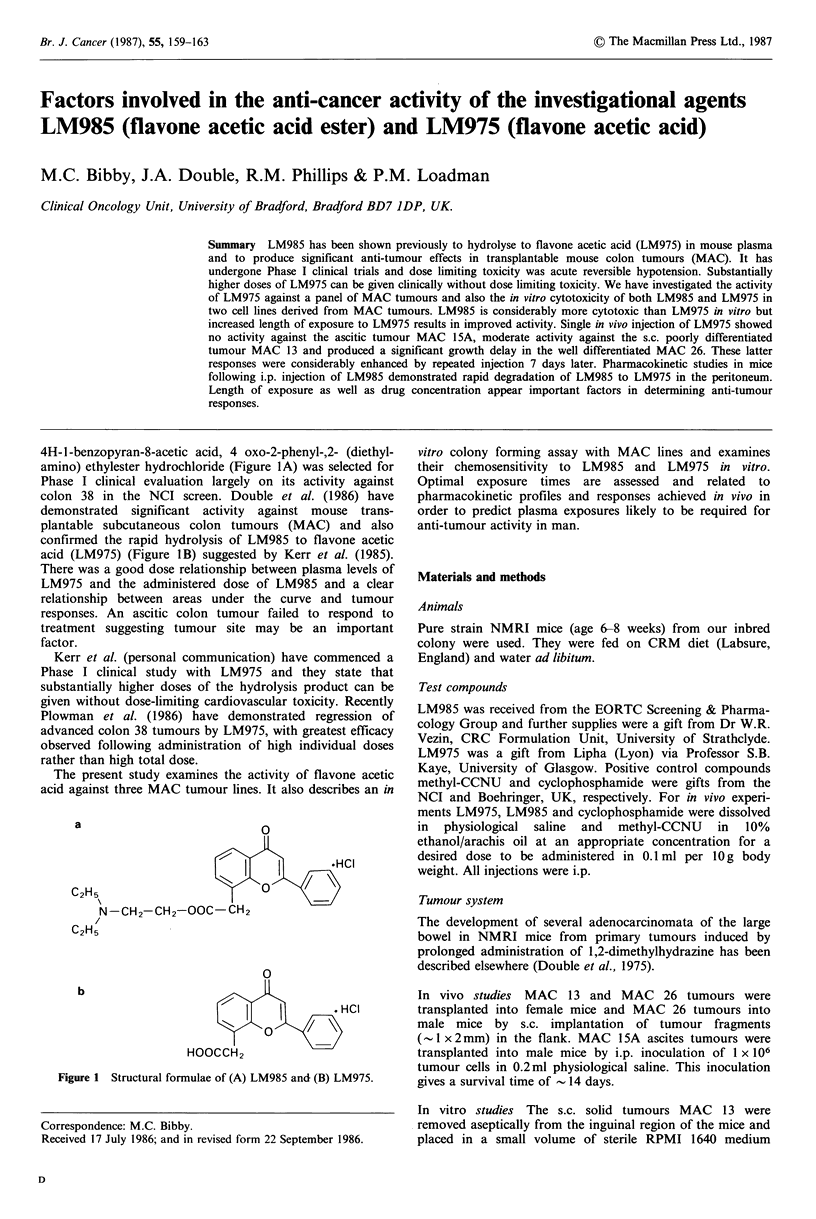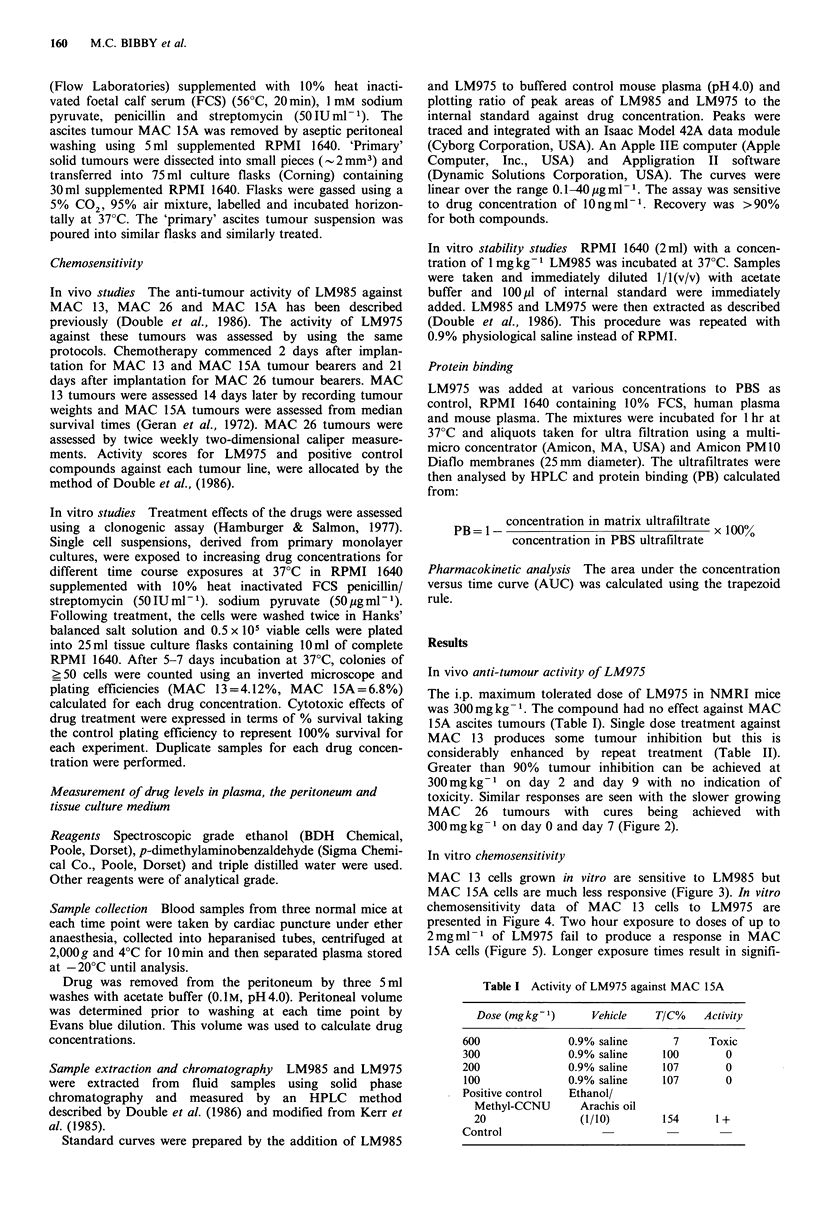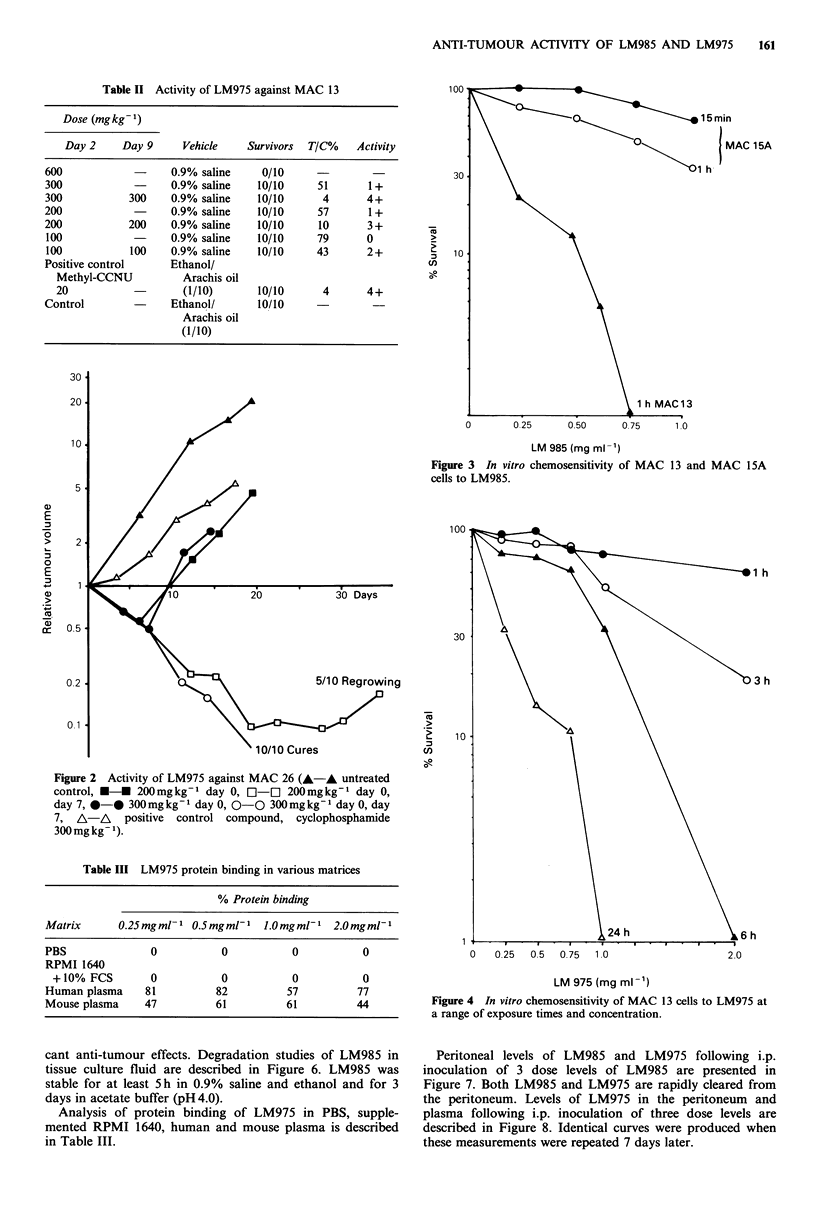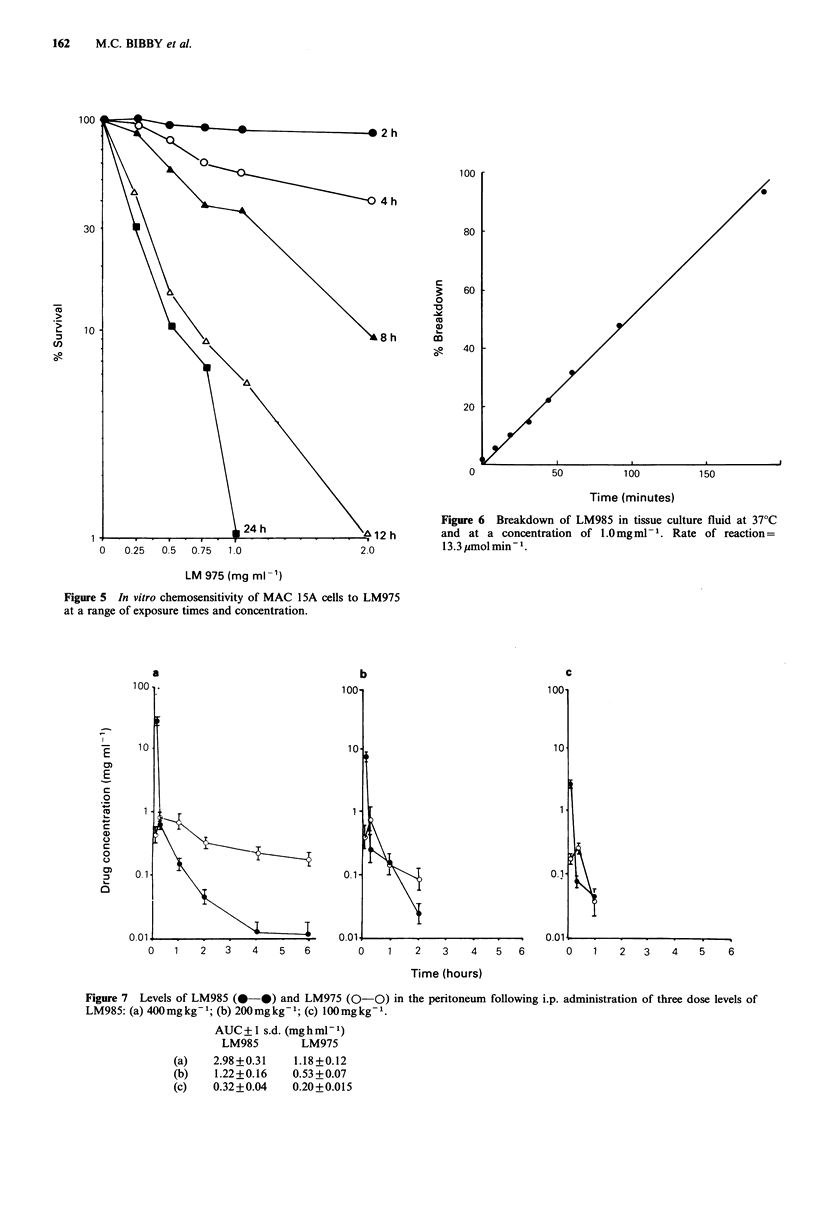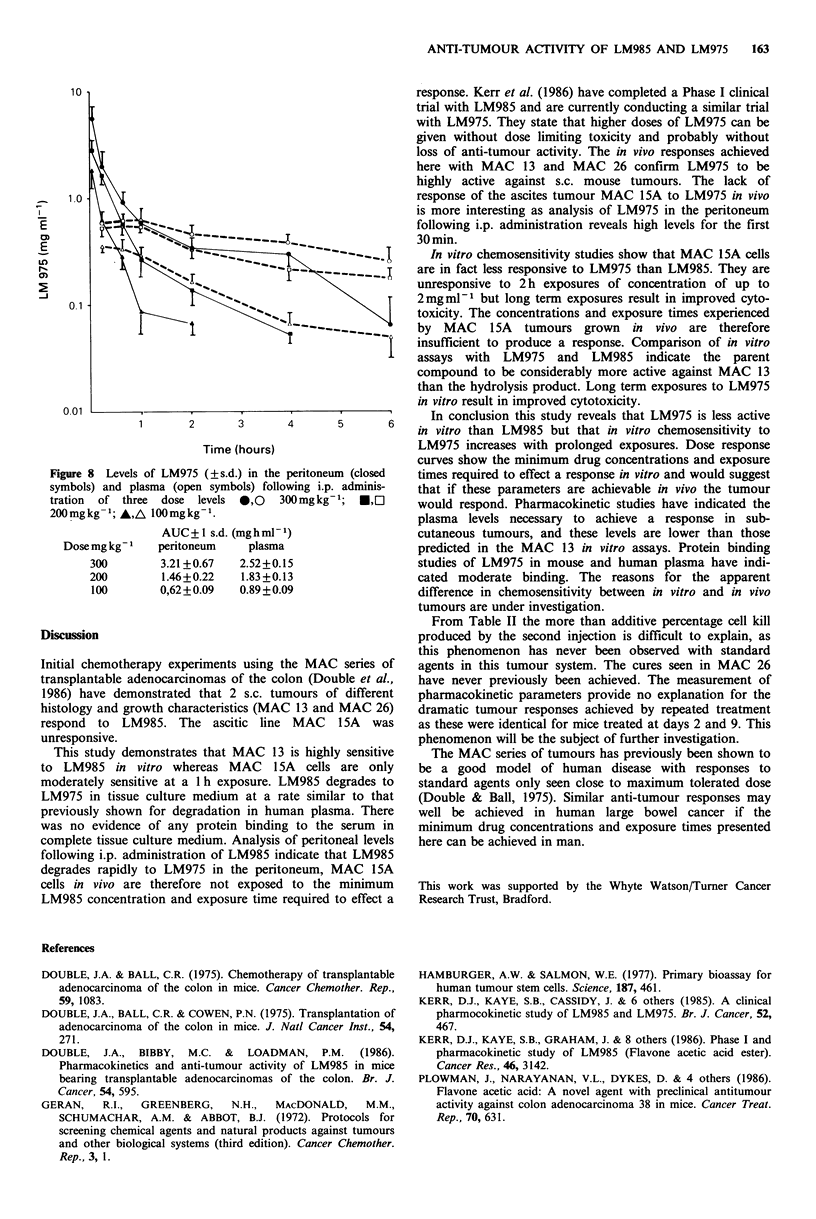# Factors involved in the anti-cancer activity of the investigational agents LM985 (flavone acetic acid ester) and LM975 (flavone acetic acid).

**DOI:** 10.1038/bjc.1987.32

**Published:** 1987-02

**Authors:** M. C. Bibby, J. A. Double, R. M. Phillips, P. M. Loadman

## Abstract

LM985 has been shown previously to hydrolyse to flavone acetic acid (LM975) in mouse plasma and to produce significant anti-tumour effects in transplantable mouse colon tumours (MAC). It has undergone Phase I clinical trials and dose limiting toxicity was acute reversible hypotension. Substantially higher doses of LM975 can be given clinically without dose limiting toxicity. We have investigated the activity of LM975 against a panel of MAC tumours and also the in vitro cytotoxicity of both LM985 and LM975 in two cell lines derived from MAC tumours. LM985 is considerably more cytotoxic than LM975 in vitro but increased length of exposure to LM975 results in improved activity. Single in vivo injection of LM975 showed no activity against the ascitic tumour MAC 15A, moderate activity against the s.c. poorly differentiated tumour MAC 13 and produced a significant growth delay in the well differentiated MAC 26. These latter responses were considerably enhanced by repeated injection 7 days later. Pharmacokinetic studies in mice following i.p. injection of LM985 demonstrated rapid degradation of LM985 to LM975 in the peritoneum. Length of exposure as well as drug concentration appear important factors in determining anti-tumour responses.


*Br. J. Cancer* (1987), **55**, 159–163 © The Macmillan Press Ltd., 1987

### Factors involved in the anti-cancer activity of the investigational agents LM985 (flavone acetic acid ester) and LM975 (flavone acetic acid)

M.C. Bibby, J.A. Double, R.M. Phillips & P.M. Loadman

*Clinical Oncology Unit, University of Bradford, Bradford BD7 1DP, UK.*

**Summary** LM985 has been shown previously to hydrolyse to flavone acetic acid (LM975) in mouse plasma and to produce significant anti-tumour effects in transplantable mouse colon tumours (MAC). It has undergone Phase I clinical trials and dose limiting toxicity was acute reversible hypotension. Substantially higher doses of LM975 can be given clinically without dose limiting toxicity. We have investigated the activity of LM975 against a panel of MAC tumours and also the *in vitro* cytotoxicity of both LM985 and LM975 in two cell lines derived from MAC tumours. LM985 is considerably more cytotoxic than LM975 *in vitro* but increased length of exposure to LM975 results in improved activity. Single *in vivo* injection of LM975 showed no activity against the ascitic tumour MAC 15A, moderate activity against the s.c. poorly differentiated tumour MAC 13 and produced a significant growth delay in the well differentiated MAC 26. These latter responses were considerably enhanced by repeated injection 7 days later. Pharmacokinetic studies in mice following i.p. injection of LM985 demonstrated rapid degradation of LM985 to LM975 in the peritoneum. Length of exposure as well as drug concentration appear important factors in determining anti-tumour responses.

4H-1-benzopyran-8-acetic acid, 4 oxo-2-phenyl-,2- (diethylamino) ethylester hydrochloride (Figure 1A) was selected for Phase I clinical evaluation largely on its activity against colon 38 in the NCI screen. Double *et al.* (1986) have demonstrated significant activity against mouse transplantable subcutaneous colon tumours (MAC) and also confirmed the rapid hydrolysis of LM985 to flavone acetic acid (LM975) (Figure 1B) suggested by Kerr *et al.* (1985). There was a good dose relationship between plasma levels of LM975 and the administered dose of LM985 and a clear relationship between areas under the curve and tumour responses. An ascitic colon tumour failed to respond to treatment suggesting tumour site may be an important factor.

Kerr *et al.* (personal communication) have commenced a Phase I clinical study with LM975 and they state that substantially higher doses of the hydrolysis product can be given without dose-limiting cardiovascular toxicity. Recently Plowman *et al.* (1986) have demonstrated regression of advanced colon 38 tumours by LM975, with greatest efficacy observed following administration of high individual doses rather than high total dose.

The present study examines the activity of flavone acetic acid against three MAC tumour lines. It also describes an *in vitro* colony forming assay with MAC lines and examines their chemosensitivity to LM985 and LM975 *in vitro*. Optimal exposure times are assessed and related to pharmacokinetic profiles and responses achieved *in vivo* in order to predict plasma exposures likely to be required for anti-tumour activity in man.

**Figure 1** Structural formulae of (A) LM985 and (B) LM975.

Correspondence: M.C. Bibby.
Received 17 July 1986; and in revised form 22 September 1986.

### Materials and methods

### *Animals*

Pure strain NMRI mice (age 6–8 weeks) from our inbred colony were used. They were fed on CRM diet (Labsure, England) and water *ad libitum*.

### *Test compounds*

LM985 was received from the EORTC Screening & Pharmacology Group and further supplies were a gift from Dr W.R. Vezin, CRC Formulation Unit, University of Strathclyde. LM975 was a gift from Lipha (Lyon) via Professor S.B. Kaye, University of Glasgow. Positive control compounds methyl-CCNU and cyclophosphamide were gifts from the NCI and Boehringer, UK, respectively. For *in vivo* experiments LM975, LM985 and cyclophosphamide were dissolved in physiological saline and methyl-CCNU in 10% ethanol/arachis oil at an appropriate concentration for a desired dose to be administered in 0.1 ml per 10 g body weight. All injections were i.p.

### *Tumour system*

The development of several adenocarcinomata of the large bowel in NMRI mice from primary tumours induced by prolonged administration of 1,2-dimethylhydrazine has been described elsewhere (Double *et al.*, 1975).

In vivo *studies* MAC 13 and MAC 26 tumours were transplanted into female mice and MAC 26 tumours into male mice by s.c. implantation of tumour fragments (~1 × 2 mm) in the flank. MAC 15A ascites tumours were transplanted into male mice by i.p. inoculation of $1 \times 10^6$ tumour cells in 0.2 ml physiological saline. This inoculation gives a survival time of ~14 days.

In vitro *studies* The s.c. solid tumours MAC 13 were removed aseptically from the inguinal region of the mice and placed in a small volume of sterile RPMI 1640 medium

(Flow Laboratories) supplemented with 10% heat inactivated foetal calf serum (FCS) (56°C, 20 min), 1 mM sodium pyruvate, penicillin and streptomycin (50 IU ml$^{-1}$). The ascites tumour MAC 15A was removed by aseptic peritoneal washing using 5 ml supplemented RPMI 1640. 'Primary' solid tumours were dissected into small pieces (~2 mm$^3$) and transferred into 75 ml culture flasks (Corning) containing 30 ml supplemented RPMI 1640. Flasks were gassed using a 5% $CO_2$, 95% air mixture, labelled and incubated horizontally at 37°C. The 'primary' ascites tumour suspension was poured into similar flasks and similarly treated.

*Chemosensitivity*

In vivo *studies* The anti-tumour activity of LM985 against MAC 13, MAC 26 and MAC 15A has been described previously (Double *et al.*, 1986). The activity of LM975 against these tumours was assessed by using the same protocols. Chemotherapy commenced 2 days after implantation for MAC 13 and MAC 15A tumour bearers and 21 days after implantation for MAC 26 tumour bearers. MAC 13 tumours were assessed 14 days later by recording tumour weights and MAC 15A tumours were assessed from median survival times (Geran *et al.*, 1972). MAC 26 tumours were assessed by twice weekly two-dimensional caliper measurements. Activity scores for LM975 and positive control compounds against each tumour line, were allocated by the method of Double *et al.*, (1986).

In vitro *studies* Treatment effects of the drugs were assessed using a clonogenic assay (Hamburger & Salmon, 1977). Single cell suspensions, derived from primary monolayer cultures, were exposed to increasing drug concentrations for different time course exposures at 37°C in RPMI 1640 supplemented with 10% heat inactivated FCS penicillin/streptomycin (50 IU ml$^{-1}$). sodium pyruvate (50 $\mu$g ml$^{-1}$). Following treatment, the cells were washed twice in Hanks' balanced salt solution and $0.5 \times 10^5$ viable cells were plated into 25 ml tissue culture flasks containing 10 ml of complete RPMI 1640. After 5–7 days incubation at 37°C, colonies of $\geqq 50$ cells were counted using an inverted microscope and plating efficiencies (MAC 13 = 4.12%, MAC 15A = 6.8%) calculated for each drug concentration. Cytotoxic effects of drug treatment were expressed in terms of % survival taking the control plating efficiency to represent 100% survival for each experiment. Duplicate samples for each drug concentration were performed.

*Measurement of drug levels in plasma, the peritoneum and tissue culture medium*

*Reagents* Spectroscopic grade ethanol (BDH Chemical, Poole, Dorset), *p*-dimethylaminobenzaldehyde (Sigma Chemical Co., Poole, Dorset) and triple distilled water were used. Other reagents were of analytical grade.

*Sample collection* Blood samples from three normal mice at each time point were taken by cardiac puncture under ether anaesthesia, collected into heparanised tubes, centrifuged at 2,000 *g* and 4°C for 10 min and then separated plasma stored at −20°C until analysis.

Drug was removed from the peritoneum by three 5 ml washes with acetate buffer (0.1 M, pH 4.0). Peritoneal volume was determined prior to washing at each time point by Evans blue dilution. This volume was used to calculate drug concentrations.

*Sample extraction and chromatography* LM985 and LM975 were extracted from fluid samples using solid phase chromatography and measured by an HPLC method described by Double *et al.* (1986) and modified from Kerr *et al.* (1985).

Standard curves were prepared by the addition of LM985 and LM975 to buffered control mouse plasma (pH 4.0) and plotting ratio of peak areas of LM985 and LM975 to the internal standard against drug concentration. Peaks were traced and integrated with an Isaac Model 42A data module (Cyborg Corporation, USA). An Apple IIE computer (Apple Computer, Inc., USA) and Appligration II software (Dynamic Solutions Corporation, USA). The curves were linear over the range 0.1–40 $\mu$g ml$^{-1}$. The assay was sensitive to drug concentration of 10 ng ml$^{-1}$. Recovery was >90% for both compounds.

In vitro *stability studies* RPMI 1640 (2 ml) with a concentration of 1 mg kg$^{-1}$ LM985 was incubated at 37°C. Samples were taken and immediately diluted 1/1(v/v) with acetate buffer and 100 $\mu$l of internal standard were immediately added. LM985 and LM975 were then extracted as described (Double *et al.*, 1986). This procedure was repeated with 0.9% physiological saline instead of RPMI.

*Protein binding*

LM975 was added at various concentrations to PBS as control, RPMI 1640 containing 10% FCS, human plasma and mouse plasma. The mixtures were incubated for 1 hr at 37°C and aliquots taken for ultra filtration using a multimicro concentrator (Amicon, MA, USA) and Amicon PM10 Diaflo membranes (25 mm diameter). The ultrafiltrates were then analysed by HPLC and protein binding (PB) calculated from:

$$PB = 1 - \frac{\text{concentration in matrix ultrafiltrate}}{\text{concentration in PBS ultrafiltrate}} \times 100\%$$

*Pharmacokinetic analysis* The area under the concentration versus time curve (AUC) was calculated using the trapezoid rule.

### Results

In vivo *anti-tumour activity of LM975*

The i.p. maximum tolerated dose of LM975 in NMRI mice was 300 mg kg$^{-1}$. The compound had no effect against MAC 15A ascites tumours (Table I). Single dose treatment against MAC 13 produces some tumour inhibition but this is considerably enhanced by repeat treatment (Table II). Greater than 90% tumour inhibition can be achieved at 300 mg kg$^{-1}$ on day 2 and day 9 with no indication of toxicity. Similar responses are seen with the slower growing MAC 26 tumours with cures being achieved with 300 mg kg$^{-1}$ on day 0 and day 7 (Figure 2).

In vitro *chemosensitivity*

MAC 13 cells grown *in vitro* are sensitive to LM985 but MAC 15A cells are much less responsive (Figure 3). *In vitro* chemosensitivity data of MAC 13 cells to LM975 are presented in Figure 4. Two hour exposure to doses of up to 2 mg ml$^{-1}$ of LM975 fail to produce a response in MAC 15A cells (Figure 5). Longer exposure times result in signifi-

**Table I** Activity of LM975 against MAC 15A

| *Dose (mg kg$^{-1}$)* | *Vehicle* | *T/C%* | *Activity* |
|---|---|---|---|
| 600 | 0.9% saline | 7 | Toxic |
| 300 | 0.9% saline | 100 | 0 |
| 200 | 0.9% saline | 107 | 0 |
| 100 | 0.9% saline | 107 | 0 |
| Positive control | Ethanol/ | | |
| Methyl-CCNU | Arachis oil | | |
| 20 | (1/10) | 154 | 1+ |
| Control | — | — | — |

**Table II** Activity of LM975 against MAC 13

| *Dose (mg kg⁻¹)* | | | | | |
|---|---|---|---|---|---|
| *Day 2* | *Day 9* | *Vehicle* | *Survivors* | *T/C%* | *Activity* |
| 600 | — | 0.9% saline | 0/10 | — | — |
| 300 | — | 0.9% saline | 10/10 | 51 | 1+ |
| 300 | 300 | 0.9% saline | 10/10 | 4 | 4+ |
| 200 | — | 0.9% saline | 10/10 | 57 | 1+ |
| 200 | 200 | 0.9% saline | 10/10 | 10 | 3+ |
| 100 | — | 0.9% saline | 10/10 | 79 | 0 |
| 100 | 100 | 0.9% saline | 10/10 | 43 | 2+ |
| Positive control Methyl-CCNU 20 | — | Ethanol/ Arachis oil (1/10) | 10/10 | 4 | 4+ |
| Control | — | Ethanol/ Arachis oil (1/10) | 10/10 | — | — |

**Figure 2** Activity of LM975 against MAC 26 (▲—▲ untreated control, ■—■ 200 mg kg⁻¹ day 0, □—□ 200 mg kg⁻¹ day 0, day 7, ●—● 300 mg kg⁻¹ day 0, ○—○ 300 mg kg⁻¹ day 0, day 7, △—△ positive control compound, cyclophosphamide 300 mg kg⁻¹).

**Table III** LM975 protein binding in various matrices

| | *% Protein binding* | | | |
|---|---|---|---|---|
| *Matrix* | *0.25 mg ml⁻¹* | *0.5 mg ml⁻¹* | *1.0 mg ml⁻¹* | *2.0 mg ml⁻¹* |
| PBS | 0 | 0 | 0 | 0 |
| RPMI 1640 +10% FCS | 0 | 0 | 0 | 0 |
| Human plasma | 81 | 82 | 57 | 77 |
| Mouse plasma | 47 | 61 | 61 | 44 |

**Figure 3** *In vitro* chemosensitivity of MAC 13 and MAC 15A cells to LM985.

**Figure 4** *In vitro* chemosensitivity of MAC 13 cells to LM975 at a range of exposure times and concentration.

cant anti-tumour effects. Degradation studies of LM985 in tissue culture fluid are described in Figure 6. LM985 was stable for at least 5 h in 0.9% saline and ethanol and for 3 days in acetate buffer (pH 4.0).

Analysis of protein binding of LM975 in PBS, supplemented RPMI 1640, human and mouse plasma is described in Table III.

Peritoneal levels of LM985 and LM975 following i.p. inoculation of 3 dose levels of LM985 are presented in Figure 7. Both LM985 and LM975 are rapidly cleared from the peritoneum. Levels of LM975 in the peritoneum and plasma following i.p. inoculation of three dose levels are described in Figure 8. Identical curves were produced when these measurements were repeated 7 days later.

**Figure 5** *In vitro* chemosensitivity of MAC 15A cells to LM975 at a range of exposure times and concentration.

**Figure 6** Breakdown of LM985 in tissue culture fluid at 37°C and at a concentration of 1.0 mg ml$^{-1}$. Rate of reaction = 13.3 μmol min$^{-1}$.

**Figure 7** Levels of LM985 (●—●) and LM975 (○—○) in the peritoneum following i.p. administration of three dose levels of LM985: (a) 400 mg kg$^{-1}$; (b) 200 mg kg$^{-1}$; (c) 100 mg kg$^{-1}$.

| | AUC ± 1 s.d. (mg h ml$^{-1}$) LM985 | LM975 |
|---|---|---|
| (a) | 2.98 ± 0.31 | 1.18 ± 0.12 |
| (b) | 1.22 ± 0.16 | 0.53 ± 0.07 |
| (c) | 0.32 ± 0.04 | 0.20 ± 0.015 |

**Figure 8** Levels of LM975 (±s.d.) in the peritoneum (closed symbols) and plasma (open symbols) following i.p. administration of three dose levels ●,○ 300 mg kg$^{-1}$; ■,□ 200 mg kg$^{-1}$; ▲,△ 100 mg kg$^{-1}$.

| Dose mg kg$^{-1}$ | AUC±1 s.d. (mg h ml$^{-1}$) peritoneum | plasma |
|---|---|---|
| 300 | 3.21±0.67 | 2.52±0.15 |
| 200 | 1.46±0.22 | 1.83±0.13 |
| 100 | 0,62±0.09 | 0.89±0.09 |

### Discussion

Initial chemotherapy experiments using the MAC series of transplantable adenocarcinomas of the colon (Double *et al.*, 1986) have demonstrated that 2 s.c. tumours of different histology and growth characteristics (MAC 13 and MAC 26) respond to LM985. The ascitic line MAC 15A was unresponsive.

This study demonstrates that MAC 13 is highly sensitive to LM985 *in vitro* whereas MAC 15A cells are only moderately sensitive at a 1 h exposure. LM985 degrades to LM975 in tissue culture medium at a rate similar to that previously shown for degradation in human plasma. There was no evidence of any protein binding to the serum in complete tissue culture medium. Analysis of peritoneal levels following i.p. administration of LM985 indicate that LM985 degrades rapidly to LM975 in the peritoneum, MAC 15A cells *in vivo* are therefore not exposed to the minimum LM985 concentration and exposure time required to effect a response. Kerr *et al.* (1986) have completed a Phase I clinical trial with LM985 and are currently conducting a similar trial with LM975. They state that higher doses of LM975 can be given without dose limiting toxicity and probably without loss of anti-tumour activity. The *in vivo* responses achieved here with MAC 13 and MAC 26 confirm LM975 to be highly active against s.c. mouse tumours. The lack of response of the ascites tumour MAC 15A to LM975 *in vivo* is more interesting as analysis of LM975 in the peritoneum following i.p. administration reveals high levels for the first 30 min.

*In vitro* chemosensitivity studies show that MAC 15A cells are in fact less responsive to LM975 than LM985. They are unresponsive to 2 h exposures of concentration of up to 2 mg ml$^{-1}$ but long term exposures result in improved cytotoxicity. The concentrations and exposure times experienced by MAC 15A tumours grown *in vivo* are therefore insufficient to produce a response. Comparison of *in vitro* assays with LM975 and LM985 indicate the parent compound to be considerably more active against MAC 13 than the hydrolysis product. Long term exposures to LM975 *in vitro* result in improved cytotoxicity.

In conclusion this study reveals that LM975 is less active *in vitro* than LM985 but that *in vitro* chemosensitivity to LM975 increases with prolonged exposures. Dose response curves show the minimum drug concentrations and exposure times required to effect a response *in vitro* and would suggest that if these parameters are achievable *in vivo* the tumour would respond. Pharmacokinetic studies have indicated the plasma levels necessary to achieve a response in subcutaneous tumours, and these levels are lower than those predicted in the MAC 13 *in vitro* assays. Protein binding studies of LM975 in mouse and human plasma have indicated moderate binding. The reasons for the apparent difference in chemosensitivity between *in vitro* and *in vivo* tumours are under investigation.

From Table II the more than additive percentage cell kill produced by the second injection is difficult to explain, as this phenomenon has never been observed with standard agents in this tumour system. The cures seen in MAC 26 have never previously been achieved. The measurement of pharmacokinetic parameters provide no explanation for the dramatic tumour responses achieved by repeated treatment as these were identical for mice treated at days 2 and 9. This phenomenon will be the subject of further investigation.

The MAC series of tumours has previously been shown to be a good model of human disease with responses to standard agents only seen close to maximum tolerated dose (Double & Ball, 1975). Similar anti-tumour responses may well be achieved in human large bowel cancer if the minimum drug concentrations and exposure times presented here can be achieved in man.

This work was supported by the Whyte Watson/Turner Cancer Research Trust, Bradford.

### References

DOUBLE, J.A. & BALL, C.R. (1975). Chemotherapy of transplantable adenocarcinoma of the colon in mice. *Cancer Chemother. Rep.*, **59**, 1083.

DOUBLE, J.A., BALL, C.R. & COWEN, P.N. (1975). Transplantation of adenocarcinoma of the colon in mice. *J. Natl Cancer Inst.*, **54**, 271.

DOUBLE, J.A., BIBBY, M.C. & LOADMAN, P.M. (1986). Pharmacokinetics and anti-tumour activity of LM985 in mice bearing transplantable adenocarcinomas of the colon. *Br. J. Cancer*, **54**, 595.

GERAN, R.I., GREENBERG, N.H., MACDONALD, M.M., SCHUMACHAR, A.M. & ABBOT, B.J. (1972). Protocols for screening chemical agents and natural products against tumours and other biological systems (third edition). *Cancer Chemother. Rep.*, **3**, 1.

HAMBURGER, A.W. & SALMON, W.E. (1977). Primary bioassay for human tumour stem cells. *Science*, **187**, 461.

KERR, D.J., KAYE, S.B., CASSIDY, J. & 6 others (1985). A clinical pharmocokinetic study of LM985 and LM975. *Br. J. Cancer*, **52**, 467.

KERR, D.J., KAYE, S.B., GRAHAM, J. & 8 others (1986). Phase I and pharmacokinetic study of LM985 (Flavone acetic acid ester). *Cancer Res.*, **46**, 3142.

PLOWMAN, J., NARAYANAN, V.L., DYKES, D. & 4 others (1986). Flavone acetic acid: A novel agent with preclinical antitumour activity against colon adenocarcinoma 38 in mice. *Cancer Treat. Rep.*, **70**, 631.